# Evaluation of Phototoxic and Skin Sensitization Potentials of PLA_**2**_-Free Bee Venom

**DOI:** 10.1155/2015/157367

**Published:** 2015-08-10

**Authors:** Yunwi Heo, Min-Jung Pyo, Seong Kyeong Bae, Hyunkyoung Lee, Young Chul Kwon, Je Hein Kim, Bokyung Kim, Choul Goo Kim, Changkeun Kang, Euikyung Kim

**Affiliations:** ^1^College of Veterinary Medicine, Gyeongsang National University, Jinju 660-701, Republic of Korea; ^2^Institute of Animal Medicine, Gyeongsang National University, Jinju 660-701, Republic of Korea; ^3^Toxicity Screening Research Center, Gyeongnam Department of Environmental Toxicology and Chemistry, Korea Institute of Toxicology (KIT), Jinju 660-844, Republic of Korea; ^4^Chung Jin Biotech Co., Ltd., Hanyang University Business Center, Anshan-si 426-791, Republic of Korea; ^5^Institutes of Agriculture and Life Science, Gyeongsang National University, Jinju 660-701, Republic of Korea

## Abstract

Bee venom (BV) from honey bee (*Apis mellifera L*.) has been used in oriental medicine and cosmetic ingredients because of its diverse pharmacological activities. In many studies, among BV components, phospholipase A_2_ (PLA_2_) is known as a major player in BV-induced allergic reaction. Therefore, we removed PLA_2_ from BV using ultrafiltration and then investigated *in vitro* phototoxicity and *in vivo* skin sensitization of PLA_2_-free BV (PBV) in comparison with regular BV. The 3T3 neutral red uptake phototoxicity assay can be appropriated to identify the phototoxic effect of a test substance upon the exposure of ultraviolet A. Chlorpromazine, a positive control, showed high levels of photoirritation factor and mean photo effect values, while BV and PBV had less of these values. Local lymph node assay is an alternative method to evaluate skin sensitization potential of chemicals. BALB/c mice were treated with *p*-phenylenediamine (PPD, positive control), BV, or PBV. In all of PPD concentrations, stimulation indexes (SI) as sensitizing potential of chemicals were ≥1.6, determined to be sensitizer, while SI levels of BV and PBV were below 1.6. Thus, based on these findings, we propose that both BV and PBV are nonphototoxic compounds and nonsensitizers.

## 1. Introduction

Bee venom (BV) from honey bee (*Apis mellifera L*.) is a complex mixture of active peptides and various enzymes, such as melittin, phospholipase A_2_ (PLA_2_), apamin, adolapin protease-inhibitors, bioactive amines, and mast cell degranulating peptide [1, 2]. It has been used in Korea, China, and Japan as a traditional medicine for acne, rheumatoid arthritis, and idiopathic Parkinson's disease [3, 4]. Recently, BV has been utilized as a cosmetic ingredient in antiaging, anti-inflammatory, and antibacterial products, due to its pharmacological effects. Nevertheless, BV can sometimes cause adverse reactions like anaphylactic shock, edema, and allergy. PLA_2_ is one of the major allergens in BV and hydrolyzes the sn-2 ester bond at membrane phospholipids configuration of free fatty acids and lysophospholipids [5, 6]. This enzyme is known to be a modulator of the release of arachidonic acid and in the synthesis of eicosanoids, which are potent inflammatory mediators [7]. Hence, we eliminated PLA_2_ from BV (PBV) using ultrafiltration methods and then investigated any potential effect of* in vitro* phototoxicity and* in vivo* skin sensitization of PBV as compared to BV [8].

Recently, several countries centered around have actively studied replacing animal experiments, on account of rules related to animal welfare. To minimize the sacrifice of animals, and to obtain the toxicity of a number of chemicals in a short duration, the development of alternative methods to animal models are required. In particular, since 2009 the European Union (EU) has banned animal testing of cosmetic products as well as their raw materials and also prohibited the sale of cosmetics developed through animal testing.

Phototoxicity (photoirritation) refers to an inflammatory reaction caused by topical application or systemic administration of a compound, followed by exposure to light (especially ultraviolet A (UVA) light). Over the past few years, several studies have tried developing a method to assess the phototoxic potential and to attempt to find the proper cell line. Interestingly, human keratinocytes had no obvious advantage as compared to BALB/c 3T3 fibroblast cell line. According to previous studies, fibroblasts have lower toxicity and higher sensitivity than keratinocytes when exposed to UVA irradiation [9–11]. Due to this reason, neutral red uptake (NRU) phototoxicity assay using BALB/c 3T3 cell line was adopted by the Organization for Economic Co-operation and Development (OECD) in 2004, as a Test Guideline (TG) [12]. OECD recommends the use of 5 J/cm^2^ UVA in the 3T3 NRU phototoxicity assay, with the attenuation of UVB from the light source, because of its inherent cytotoxicity. In 3T3 NRU assay, phototoxicity is determined by measuring cytotoxicity of BALB/c 3T3 upon the exposure to an exogenous testing chemical, followed by subsequent presence or absence of UVA irradiation.

Local lymph node assay (LLNA) evaluates the skin sensitization potential of chemicals. This assay replaces the guinea pig maximization test (GPMT), thus providing animal welfare benefits, generates quantitative data, and reduces the test time [13]. The skin sensitization potential of a substance is usually evaluated by observing lymphocyte proliferation of auricular lymph nodes in response to the ear-treatment of the substance. LLNA-ELISA method does not require the use of radioisotope ^3^H-thymidine, but 5-bromo-2′-deoxyuridine (BrdU), which is incorporated into the DNA of proliferating lymphocytes during the S-phase of cell cycle. Hence, this assay has been approved by the Environmental Protection Agency (EPA), OECD, Interagency Coordinating Committee on the Validation of Alternative Methods (ICCVAM), and the European Centre for the Validation of Alternative Methods (ECVAM).

In this study, we used alternative methods to investigate whether BV and/or PBV can be applied as a cosmetic ingredient in skin care products. To evaluate phototoxic and skin sensitization potentials of PBV, we performed 3T3 NRU phototoxicity assay and LLNA, respectively. Consequently, our studies revealed that PBV and BV can be developed for cosmetic application because they appear to have neither phototoxicity nor sensitizing effects.

## 2. Materials and Methods

### 2.1. Chemicals and Reagents

The BALB/c 3T3 cell line was obtained from ATCC. Neutral Red, CPZ, PPD, and BrdU were obtained from Sigma-Aldrich (San Diego, CA). PPD was dissolved in acetone : olive oil (AOO; 4 : 1) and BV and PBV were dissolved in distilled water (DW). BrdU was dissolved in phosphate-buffered saline (PBS) at a concentration of 20 mg/mL. All other chemicals and reagents were obtained from Sigma-Aldrich, unless noted otherwise.

### 2.2. Segregation of PLA_2_ from Bee Venom

BV and PBV prepared from honey bee are supplied from Chung Jin Biotech, Korea. Briefly, BV was collected using bee venom collector (Chung Jin Biotech, Ashan-si, Korea) in a sterile manner under strict laboratory conditions. BV collector was placed on the hive, and bees were given electronic shocks to cause them to sting onto a glass plate of BV collector from which dried BV was later scraped off. Collected BV was dissolved in DW, and then it was filtered using 3.0 *μ*m filtration membrane to remove big debris like dust and pollen. Subsequently, 0.45 and 0.2 *μ*m membrane filters were used to eliminate, if any, tiny debris. The filtered BV was lyophilized and stored at −20°C for later use. PBV was prepared according to the following procedures. The filtration was conducted using a stirred ultrafiltration (Millipore series 8400, Merck kGaA, Darmstadt, Germany) cell with a 10 kDa molecular weight cut-off membrane (Ultracel PL regenerated cellulose, 76 mm, Millipore Corporation, Bedford, MA, USA). PBV obtained was lyophilized and stored at −20°C for later use.

### 2.3. *In Vitro* 3T3 NRU Phototoxicity Test

#### 2.3.1. Cell Culture

BALB/c 3T3 cells were cultivated in Dulbecco's Modified Eagle Medium (DMEM) which contained 10% newborn calf serum (NCS), 4 mM glutamine, and 1% penicillin/streptomycin.

#### 2.3.2. 3T3 NRU Phototoxicity Test

This assay was performed according to the OECD guideline [12]. BALB/c 3T3 cells were seeded (1 × 10^4^ cells/well) into two plates using 96-well culture plate. After 24 h, the medium was changed to free phenol red DMEM without NCS. Eight concentrations of test compound (0~22.5 ug/mL for CPZ, 0~1 ug/mL for BV and PBV) were treated in both plates and incubated for 1 h, respectively. After 1 h, medium was changed into free phenol red DMEM without NCS. One of plates was put in UVA irradiation environment (+UVA) and the other plate was put in dark warm place (−UVA). +UVA manufactured 365 nm and plates were exposed for 28 min to light at 2.97 mW/cm^2^ (= 5 J/cm^2^) (BLX 365, Bio-Link, France). All of wells were replaced with DMEM containing NCS at the end of UVA irradiation and then incubated overnight.

Neutral red powder dissolved in free phenol red DMEM without NCS (neutral red/DMEM, 50 *μ*g/mL). Two plates changed to neutral red medium and incubated for 3 h. After that, neutral red medium was discarded and cells were treated with extract solution (water : ethanol : acetic acid = 49 : 50 : 1) and put the plate on a shaker for 10 min to dissolve. Both plates were measured at a wavelength of 540 nm, using a spectrophotometric microplate reader (PowerWave XS, BioTek instruments, Inc., Winooski, VT, USA). Analyzed data were calculated using Phototox Version 2.0 software (freeware obtained from OECD Guideline). The software detected photoirritation factor (PIF) and mean photoeffect (MPE). 

### 2.4. LLNA

#### 2.4.1. Animals

Female 8-week-old BALB/c mice were purchased from Orient Bio (Seongnam-Si, Korea). All animals were used at age 9 weeks. The animals were maintained in the Laboratory Animal Research Center (LARC) of Gyeongsang National University (GNU-140212-M0009). Animals were kept under controlled conditions of temperature (23 ± 3°C) and relative humidity (50 ± 10%) with alternating 12 h light and dark cycle. The care and treatment of the mice were in accordance with the guidelines established by the Public Health Service Policy on the Human Care and Use of Laboratory Animals and were approved by the Institutional Animal Care and Use Committee. Each group included 5 animals.

#### 2.4.2. Sensitization

The LLNA was performed as described by an OECD guideline 442B [14]. Briefly, 7 groups of five mice received 25 *μ*L of either the vehicle, the positive control compound PPD (0.1, 1, and 3%), BV, or PBV (100 mg/mL, highest melting concentration), on the dorsum of both ears daily for 3 consecutive days. On day 5, the animals were injected intraperitoneally with 0.1 mL BrdU (20 mg/mL) and mice were sacrificed after 24 h (day 6). Ear thickness was measured with a micrometer (Digimatic Caliper, Mitutoyo, Japan); ear punch biopsies (6 mm full thickness skin) were collected and weighed with a laboratory balance as a marker of ear swelling. Both auricular lymph nodes were isolated and weighed and undergone lymphocyte preparation.

#### 2.4.3. ELISA for BrdU Positive Lymph Node Cells (LNCs)

LNCs were prepared from lymph node by collapse through 70 *μ*m mesh (BD Pharmingen, Franklin, NJ) in 15 mL PBS. The LNCs were counted using a hematocytometer after staining with trypan blue. After counting, LNCs (100 *μ*L) were seeded into 96-well plates and centrifugation (300 ×g) was for 10 min. Subsequently, LNCs were fixed and permeabilized, according to the instruction manual of ELISA kits (Roche Applied Science, Mannheim, Germany). The absorbance was measured at a wavelength of 370 nm, using a spectrophotometric microplate reader.

### 2.5. Calculations of Stimulation Indices (SI)

The skin sensitizing potential of a test substance is indicated by an increase in the SI. SI is the ratio of the mean BrdU labeling index for each treatment group to the mean BrdU labeling index of the concurrent vehicle control group. The SI of 1.6 indicates a positive threshold response in the assay.

### 2.6. Statistical Analysis

The results are expressed as a mean ± standard deviation (SD). A paired Student's* t-*test was used to assess the significance of differences between two mean values. *P* < 0.05 was considered to be statistically significant.

## 3. Results

### 3.1. Phototoxic Effects of BV

Our previous study showed that the original BV has strong PLA_2_ activity, while PBV retained only insignificant enzyme activity.

**Figure 1:**
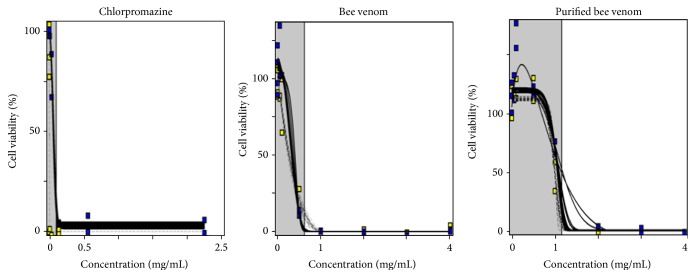
Phototoxicity of test compound in the 3T3 NRU PT. The 3T3 cells were treated with different concentration of CPZ, BV, and PBV with UVA light (5 J/cm^2^). Data represent mean ± SD of three experiments.

The phototoxicity test was evaluated using positive control (CPZ), BV, and PBV. The BALB/c 3T3 cells were exposed to various concentration of the test materials, after which they were exposed to UVA (5 J/cm^2^) for 1 h, following which the cell viability was determined as per the NRU phototoxicity assay.  Figure 1 showed representative cell viability curves of 3T3 cells after treatment with CPZ, BV, or PBV. Using Phototox Version 2.0 software, the PIF and MPE were calculated and the data assessed (Table 1). The PIF compares the EC_50_ of the test substance (the concentration at which the compound inhibits 50% viability of the cells, as compared to control) in the presence (+UVA) and absence (−UVA) of UVA irradiation, using the following formula:

(1) $$\begin{array}{c} PIF=\frac{{EC}_{50}\left(-UVA\right)}{{EC}_{50}\left(+UVA\right)}. \end{array}$$

The MPE is defined as a weighted average across a set of individual photoeffect values and is based on the comparison of the response curves generated with and without UVA exposer. It is measured using the following formula:

(2) $$\begin{array}{c} MPE=\frac{\sum_{i=1}^{n}W_{i}{PE}_{C_{i}}}{\sum_{i=1}^{n}W_{i}}. \end{array}$$

The EC_50_ values in CPZ were 8.49 *μ*g/mL and 0.15 *μ*g/mL, without or with UVA, respectively. MPE and PIF values were 0.65 and 35.68. For acceptance criteria, CPZ of known phototoxic substance were ascertained every time. MPE and PIF for BV in 3T3 cells were evaluated at 0.11 and 2.16, respectively. Based on these results, BV was determined to be a probable phototoxic substance. Conversely, PBV was not phototoxic compound, as it had a low level of MPE and PIF (0.01 and 1.30, resp.). The EC_50_ values, with and without UVA exposure, were not very different in both BV and PBV treated cells (Table 2). These data demonstrate that both BV and PBV are nonphototoxic substances.

### 3.2. Skin Sensitization Effect of BV

**Figure 2:**
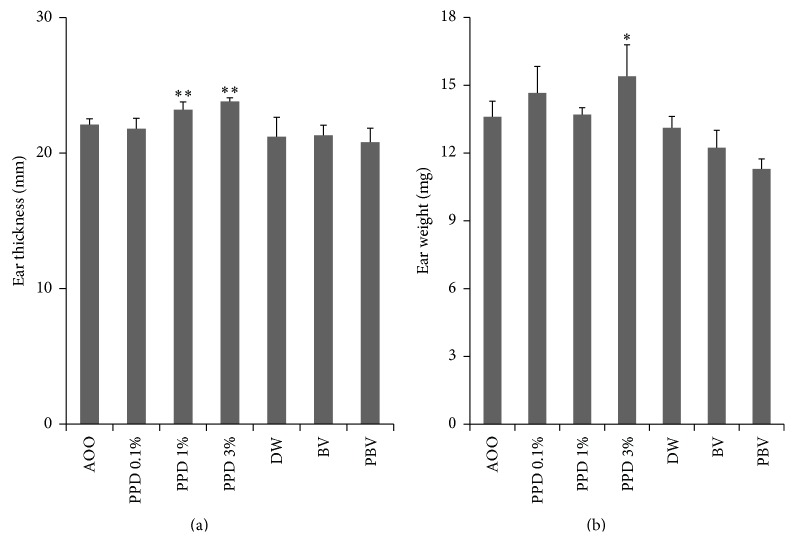
Differences in ear thickness and ear weight. (a) After mice were treated with 0.1, 1, and 3% PPD, BV, and PBV for 3 days ear thickness was measured on day 5 and weighed. (b) The ear weight of mice. 6 mm diameter of ear was monitored on day 5. Data represent mean ± SD from 5 mice, ^*∗*^ *P* < 0.05, ^*∗∗*^ *P* < 0.01 compared to the control.

**Figure 3:**
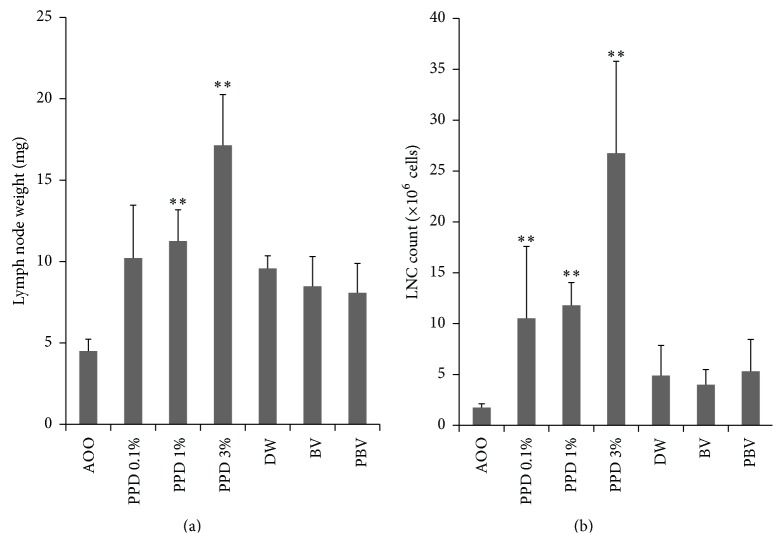
Auricular lymph node weight and the number of lymph node cells. (a) Lymph node weight indicates significant difference from the control group and applied group. (b) LNCs (lymph node cells) were measured using a hemacytometer after disassembly. Data represent mean ± SD from 5 mice, ^*∗∗*^ *P* < 0.01, compared to the vehicle group.

PPD, BV, or PBV were applied to the dorsal surface of both mouse ears. After 3 consecutive days of application, ear thickness and ear weight were measured (DW and AOO were used as a negative control). PPD showed a concentration dependent increase, but BV and PBV showed no change in ear thickness and weight (Figure 2). To compare changes in the lymph node, auricular lymph nodes were isolated and weighed on day 6. The auricular lymph node weights, and LNC counts, increased in a dose-dependent manner with PPD application, while BV and PBV application showed no changes (Figure 3).

**Figure 4:**
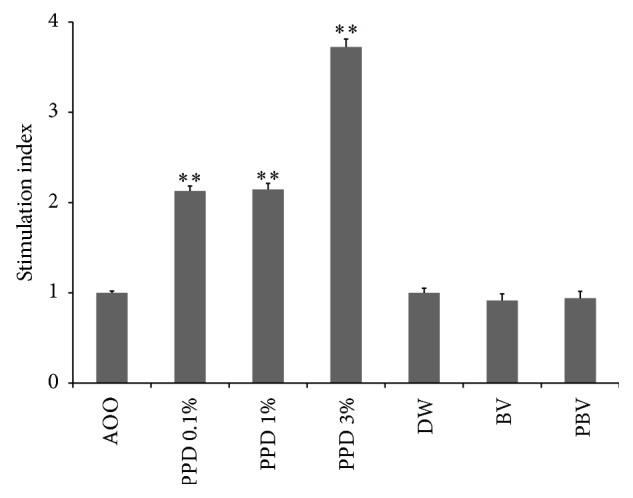
Stimulation index (SI) following BrdU ELISA in lymph nodes. SI was calculated as the ratio of the mean BrdU incorporation for each treatment group versus that of the vehicle control group. The values are in terms of mean ± SD (*n* = 5). ^*∗∗*^ *P* < 0.01.

**Figure 5:**
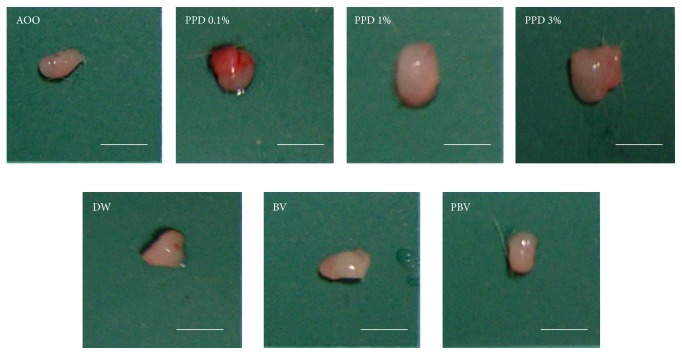
Actual sizes of representative auricular lymph node from the treated mouse. Mice were treated with 0.1, 1, and 3% PPD, BV, and PBV for 3 days. Bars indicated 3 mm.

The proliferation of the LNCs was assessed by ELISA, using the BrdU cell proliferation assay. BrdU gets incorporated into newly synthesized DNA strands of actively proliferating LNCs. The skin sensitizing potential of a test substance is indicated by an increase in the SI of BrdU incorporation by a factor of >1.6, when compared with the concurrent vehicle control group. All concentrations of the known sensitizer PPD (0.1, 1, and 3%) produced SI values >1.6. However, LNC proliferation and SI values were not affected by either BV or PBV (Figure 4). The weights of the representative auricular lymph nodes also showed the same pattern as LNCs proliferation and SI values (Figure 5). These results imply that BV and PBV could be considered as nonsensitizers.

## 4. Discussion 

Previously, BV has been widely used treatment in the past, including acne, rheumatoid arthritis, cancer, and Parkinson's disease [3, 4, 15]. These days it is also used as cosmetic ingredient. Despite these uses, BV contains PLA_2_ which is the main component of the allergen and induces adverse effects such as anaphylactic shock, inflammation, and edema. Hence, to evaluate phototoxic and skin sensitization potentials of PBV, we performed 3T3 NRU phototoxicity assay and LLNA to ensure its safety for cosmetic products.

The potential of BV and PBV as phototoxic substances was tested using 3T3 NRU phototoxicity assay. UV related toxicity is divided into 3 parts, UVA (320~400 nm), UVB (280~320 nm), and UVC (200~280 nm). The UVC hardly reaches the earth because it gets absorbed in the ozone layer of the stratosphere, whereas UVA and UVB reach the surface, thus affecting the human skin. UVA radiation exhibits its effects in pigmentation and premature aging, reduces elasticity on dermis due to the collapse of the elastic fibers, and promotes expansion of capillaries and their destruction. Therefore, the 3T3 NRU phototoxicity assay was used to evaluate toxicity of substance when exposed to UVA [16].

The assay determines the phototoxicity and photoallergenicity using neutral red. The specificity, reproducibility, and sensitivity of the 3T3 NRU phototoxicity assay were better than other assays; also, the phototoxic potential of a substance as indicated by this assay matches the outcome of phototoxicity in 95% animal experiments. It was used for neutral dye accumulation in intact cell lysosomes. Lysosomal membrane is influenced by phototoxic substances, which induce the reduction in the absorption and binding of neutral red. When substances do not absorb light of a particular wavelength in the cell, the light will be transmitted through the cell and no effect is seen. Conversely, when a substance absorbs light, it can be passed to another molecule, and the material will change itself. It can make free radicals, which affect the lipid oxidation of the cell membrane, permeability or transport ability is affected by the enzymes inactivated, metabolic dysfunction due to DNA transcription errors could occur, or it may cause incorrect decryption RNA. Previous studies demonstrated that CPZ induces DNA, RNA, cell membrane, and soluble protein changes in the cell [17]. Our present study showed that the CPZ induced phototoxicity, while BV has a low phototoxic potential. However, PBV was found to be superior to BV in phototoxicity levels. Our data demonstrated that PBV can be readily accessible and safe for use as a cosmetic ingredient (Figure 1).

We also evaluated the potency of skin sensitization of BV and PBV by murine LLNA. Many chemicals have the potential to cause contact allergic dermatitis, leading to serious health problems. The GPMT has been used to assess the sensitizing and the cross sensitizing potential between chemicals. Traditional ^3^H-thymidine LLNA method has several advantages over the GPMT, including generation of quantitative data, a shorter test time, a reduction in the number of animals, and welfare. However, ^3^H-thymidine LLNA has disadvantages such as radioactive substance injection into animals, environmental concerns, ventilation facility requirements, and necessary expenses to dispose radioactive substances. On the other hand, BrdU LLNA could provide a good alternative screening method for contact allergen because it is simple and does not use radioisotopes. Therefore, in this study, we used BrdU LLNA assay to show whether BV and PBV were sensitizers or not. OECD recommends using CBA/JN mouse strain; however, preceding studies have revealed that there was no difference in sensitivity between BALB/c and CBA/JN. Due to this reason, we used BALB/c mouse strain, which is relatively easier to obtain [18]. Results of our LLNA showed that sensitization with PPD was concentration dependent, with no change in SI values with either BV or PBV (Figure 4).

Contact sensitization is generally known to occur as the immune response through T cell mediation, although an increase of the B lymphocytes is also seen. Cytokines are activated by the T cells, causing an allergic response. Previous studies found that cytokines such as IL-2, IL-4, IL-5, and IFN-*γ* increase due to the irritant [19]. Although we did not test BV and PBV-mediated cytokine level changes, no inflammatory responses were observed.

Inflammation and allergic contact dermatitis are usually associated with various proinflammatory mediators, including nitric oxide (NO) and prostaglandin E2, which are the markers of inflammatory reactions production and the synthesis of inflammatory cytokines [20]. NO is rapidly induced when stimulated with inflammatory environments, such as lipopolysaccharides and pathogens. It is an important indicator of inflammatory response and the detection of NO level is pivotal to assess the potential anti-inflammatory effect of a test compound. Briefly, our previous study showed that the inhibitory activities on LPS-induced NO generation were determined in RAW264.7 cells. The cotreatment of PBV showed a significant suppression of NO generation. The results indicated that PBV has anti-inflammatory activity also [8]. Furthermore, it has been previously reported that in human maximization test (HMT) it is evident that the LLNA identifies those chemicals that are significant human contact allergens. In addition, LLNA specificity is better than HMT [21].

Although there was a reduction in the number of animals when compared with GPMT, LLNA still required animal experiments, in the course of which mice were sacrificed. Recently, several cell-based* in vitro* methods for skin sensitization have been proposed. A Japanese cosmetic company has developed the human cell line activation test (h-CLAT) [22]. Until now this* in vitro* assay had not been formulated. In the future, if we were to expel the use of animals, this assay will be worth evaluating.

## 5. Conclusion 

In our studies, we have evaluated 3T3 NRU phototoxicity and LLNA of BV and PBV. We can conclude by saying that BV and PBV exhibited no irritation potential in BALB/c cell line and mice. Even though BV has PLA_2_, it showed the same effect as PBV; that is, both compounds were nonphototoxic and nonsensitizers. Our results suggest that it would be safe to use both BV and PBV in cosmetic ingredients and medical applications.

## Figures and Tables

**Table 1:** 3T3 NRU phototoxicity assay validation classification based on PIF and MPE.

Interpretation	MPE	PIF
No phototoxicity	MPE < 0.1	PIF < 2
Probable phototoxicity	0.1 < MPE < 0.15	2 < PIF < 5
Phototoxicity	MPE > 0.15	PIF > 5

**Table 2:** Phototoxicity data for CPZ, BV, and PBV in BALB/c 3T3 cells.

	UVA− EC_50_ (*µ*g/mL)	UVA+ EC_50_ (*µ*g/mL)	MPE	PIF
CPZ	8.5 ± 11.2	0.2 ± 0.2	0.65 ± 0.02	35.7 ± 9.8
BV	366.0 ± 131.0	124.9 ± 117.6	0.11 ± 0.13	2.2 ± 1.4
PBV	1374.7 ± 270.6	1056.0 ± 88.8	0.01 ± 0.05	1.3 ± 0.2

Data represent mean ± SD from three independent experiments.
